# Lysosomal Ca^2+^ as a mediator of palmitate-induced lipotoxicity

**DOI:** 10.1038/s41420-023-01379-0

**Published:** 2023-03-21

**Authors:** Soo-Jin Oh, Yeseong Hwang, Kyu Yeon Hur, Myung-Shik Lee

**Affiliations:** 1 Department of Health Sciences and Technology, SAIHST, Sungkyunkwan University, Seoul, 06355 Korea; 2 Department of Integrated Biomedical Science, Soonchunhyang Institute of Medi-bio Science and Division of Endocrinology, Department of Internal Medicine, Soonchunhyang Medical Center, Soonchunhyang University College of Medicine, Cheonan, Korea; 3 Severance Biomedical Science Institute, Graduate school of Medical Science, BK21 Project, Yonsei University College of Medicine, Seoul, 03722 Korea; 4 Department of Medicine, Samsung Medical Center, Sungkyunkwan University School of Medicine, Seoul, Korea

**Keywords:** Pathogenesis, Calcium signalling

## Abstract

While the mechanism of lipotoxicity by palmitic acid (PA), an effector of metabolic stress in vitro and in vivo, has been extensively investigated, molecular details of lipotoxicity are still not fully characterized. Since recent studies reported that PA can exert lysosomal stress in addition to well-known ER and mitochondrial stress, we studied the role of lysosomal events in lipotoxicity by PA, focusing on lysosomal Ca^2+^. We found that PA induced accumulation of mitochondrial ROS and that mitochondrial ROS induced release of lysosomal Ca^2+^ due to lysosomal Ca^2+^ exit channel activation. Lysosomal Ca^2+^ release led to increased cytosolic Ca^2+^ which induced mitochondrial permeability transition (mPT). Chelation of cytoplasmic Ca^2+^ or blockade of mPT with olesoxime or decylubiquinone (DUB) suppressed lipotoxicity. Lysosomal Ca^2+^ release led to reduced lysosomal Ca^2+^ content which was replenished by ER Ca^2+^, the largest intracellular Ca^2+^ reservoir (ER → lysosome Ca^2+^ refilling), which in turn activated store-operated Ca^2+^ entry (SOCE). Inhibition of ER → lysosome Ca^2+^ refilling by blockade of ER Ca^2+^ exit channel using dantrolene or inhibition of SOCE using BTP2 inhibited lipotoxicity in vitro. Dantrolene or DUB also inhibited lipotoxic death of hepatocytes in vivo induced by administration of ethyl palmitate together with LPS. These results suggest a novel pathway of lipotoxicity characterized by mPT due to lysosomal Ca^2+^ release which was supplemented by ER → lysosome Ca^2+^ refilling and subsequent SOCE, and also suggest the potential role of modulation of ER → lysosome Ca^2+^ refilling by dantrolene or other blockers of ER Ca^2+^ exit channels in disease conditions characterized by lipotoxicity such as metabolic syndrome, diabetes, cardiomyopathy or nonalcoholic steatohepatitis.

## Introduction

Palmitic acid (PA) is an important molecule acting as an effector of lipid injury and metabolic stress in vitro and in vivo. Mechanisms of PA-induced lipotoxicity have been ascribed to endoplasmic reticulum (ER) stress, mitochondrial stress, production of ceramide or lysophosphatidylcholine [1–4], leading to JNK activation, apoptosis or necrosis, depending on the experimental condition and cellular context [3, 5–7]. While ER and mitochondria are well-established target organelles of PA-induced injury [2, 4, 8, 9], lysosomal changes have recently been reported to occur such as decreased lysosomal acidity, altered lysosomal enzyme activity or impaired lysosomal integrity [10–13]. We recently reported that lysosomal Ca^2+^ is released after treatment with PA, while the cellular consequences of lysosomal Ca^2+^ release could be distinct depending on the cell types or context of treatment [11, 12]. Since release of lysosomal Ca^2+^ can also lead to increased cytosolic Ca^2+^ content ([Ca^2+^]_i_) [11, 12] and Ca^2+^ is one of the most important inducers of mitochondrial permeability transition (mPT) and subsequent cell death [14, 15], we conducted this investigation based on our hypothesis that lysosomal Ca^2+^ release by PA can lead to lipotoxic cell death through mPT.

## Results

### PA elicits lysosomal Ca^2+^ release

PA can induce reactive oxygen species (ROS) by compromising mitochondrial complex I and III [11, 16], which can activate lysosomal Ca^2+^ channel [12, 17]. Lysosomal Ca^2+^ release due to lysosomal Ca^2+^ exit channel activation can increase cytosolic Ca^2+^ content ([Ca^2+^]_i_), which, in turn, can induce mitophagy, lysosomal stress response [11, 12] or diverse types of cell death [18–20]. Hence, we studied whether PA-induced lipotoxicity entails mitochondrial ROS-induced lysosomal Ca^2+^ release and increased [Ca^2+^]_i_.

**Fig. 1:**
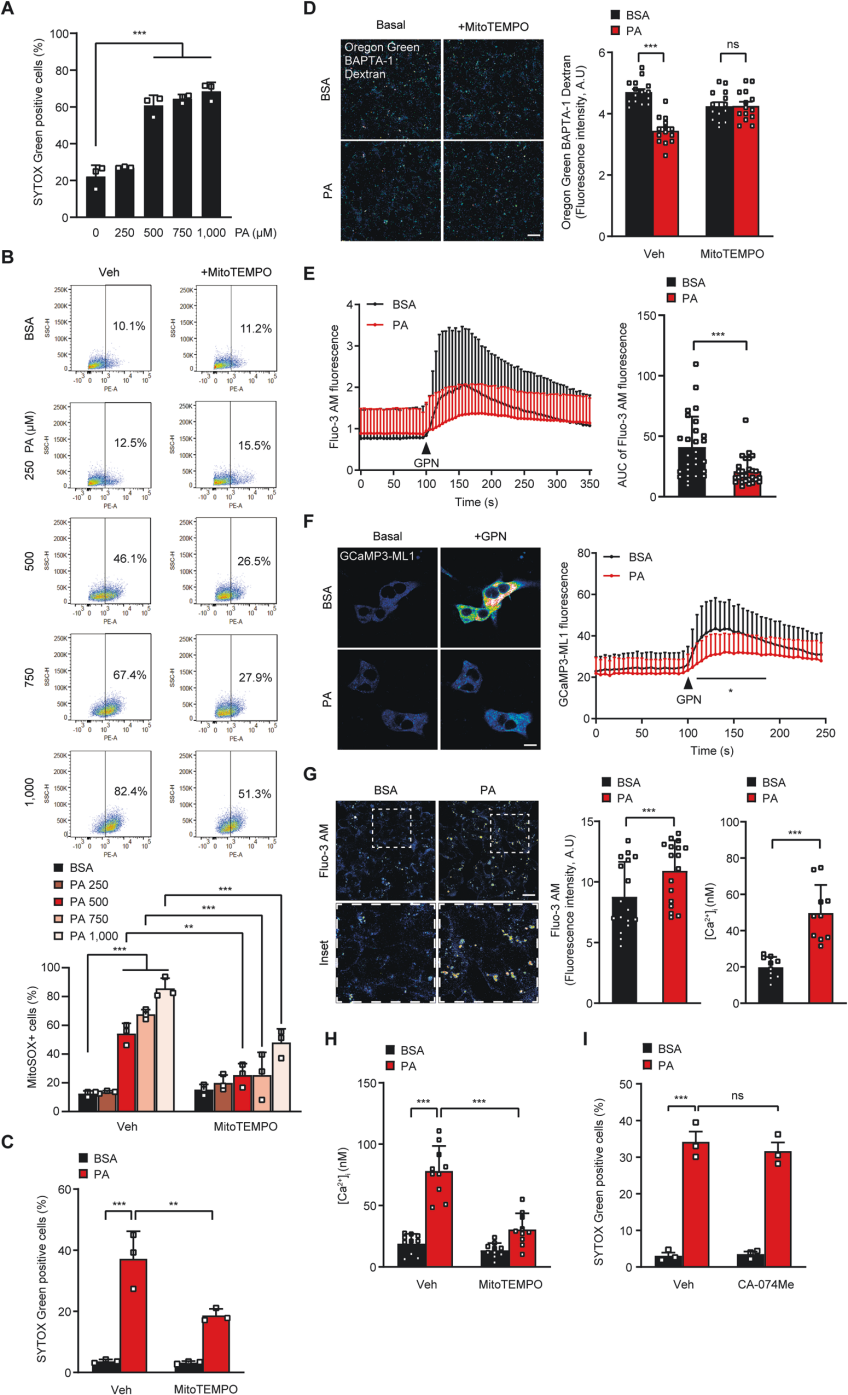
PA reduces lysosomal Ca^2+^. **A** After treatment of HepG2 cells with 250–1000 μM PA for 24 h, cell death was determined using SYTOX Green. **B** After treatment of HepG2 cells with 250–1000 μM PA for 24 h in the presence or absence of 10 μM MitoTEMPO, PA-induced mitochondrial ROS was measured by MitoSOX followed by flow cytometry (lower panel). Representative scattergrams are shown (upper panel). **C** After treatment of HepG2 cells with 500 μM PA for 24 h in the presence or absence of 10 μM MitoTEMPO, cell death was determined using SYTOX Green. **D** Cells were loaded with Oregon Green-488 BAPTA-1 Dextran (OGBD) for 16 h and chased for 4 h. Cells were then treated with PA or BSA for 6 h in the presence or absence of 10 μM MitoTEMPO, followed by confocal microscopy to determine [Ca^2+^]_Lys_ (right). Representative fluorescence images are shown (left panel). Scale bar, 20 μm. **E** After staining with Fluo-3 AM for 30 min, cells were treated in PA or BSA for 6 h, followed by confocal microscopy to monitor GPN-induced changes of lysosomal Ca^2+^ release and [Ca^2+^]_i_ increase (left). AUC of the curve was calculated as an estimate of lysosomal Ca^2+^ content (right). **F** Cells transfected with *GCaMP3-ML1* were treated with PA or BSA for 6 h, and then GPN-induced change of *GCaMP3-ML1* fluorescence was determined by confocal microscopy to visualize perilysosomal Ca^2+^ release (right). Representative fluorescence images are shown (left panel). Scale bar, 10 μm. **G** [Ca^2+^]_i_ in HepG2 cells treated with PA for 6 h was determined by staining with Fluo-3 AM (middle) or ratiometric analysis after Fura-2 loading (right). Representative Fluo-3 fluorescence images are shown (left panel). Scale bar, 20 μm. **H** [Ca^2+^]_i_ in HepG2 cells treated with PA for 6 h in the presence or absence of 10 μM MitoTEMPO was determined by ratiometric analysis after Fura-2 loading. **I** After treatment of HepG2 cells with PA in the presence or absence of 10 μM CA-074Me for 24 h, cell death was determined using SYTOX Green. Data are expressed as the means ± SD of three independent experiments. Statistical comparisons were performed using one-way ANOVA with Tukey’s multiple comparison test (**A**–**D**, **H, I**) or two-tailed unpaired Student’s *t*-test (vehicle and PA comparisons in **E**–**G**). (*P < 0.05; **P < 0.01; ***P < 0.001; ns not significant).

When we treated HepG2 cells with PA, significant lipotoxic cell death was observed in a dose range of 500 ~ 1,000 μM, as revealed by SYTOX Green staining (Fig. 1A). Furthermore, accumulation of mitochondrial ROS stained with MitoSOX was well visualized in the same dose range of PA, which was significantly reduced by MitoTEMPO, a mitochondrial ROS quencher (Fig. 1B), indicating mitochondrial ROS generation by PA. As PA doses higher than 500 μM could be unphysiologically high [21], we employed 500 μM of PA in the following experiments to minimize unphysiological effects of high dose of PA. In an experiment to find causal relationship between lipotoxic cell death and mitochondrial ROS, PA-induced death of HepG2 cells was significantly reduced by MitoTEMPO (Fig. 1C), suggesting that mitochondrial ROS generation is a critical event in PA-induced lipotoxicity. When we studied possible changes of lysosomal Ca^2+^ after PA treatment, concentration of lysosomal Ca^2+^ ([Ca^2+^]_Lys_) stained with Oregon Green-488 BAPTA-1 dextran (OGBD) was significantly decreased in HepG2 cells treated with PA (Fig. 1D), suggesting release of lysosomal Ca^2+^ by PA treatment, similar to the results using other types of cells [11, 12]. When we indirectly estimated lysosomal Ca^2+^ content by calculating area under the curve (AUC) of cytosolic Ca^2+^ concentration ([Ca^2+^]_i_) tracing using Fluo-3 AM staining after treatment with Gly-Phe β-naphthylamide (GPN), a lysosomotropic agent [22], lysosomal Ca^2+^ content was again significantly reduced by PA treatment (Fig. 1E). Decrease of [Ca^2+^]_Lys_ was abrogated by MitoTEMPO (Fig. 1D), suggesting that mitochondrial ROS produced by PA treatment induces lysosomal Ca^2+^ release. We also studied whether perilysosomal Ca^2+^ release can be observed after PA treatment employing GCaMP3-ML1, a probe detecting perilysosomal Ca^2+^ release [22]. In *GCaMP3-ML1*-transfected HepG2 cells, perilysosomal Ca^2+^ release was not directly visualized by PA treatment (Fig. 1F). However, perilysosomal Ca^2+^ release after treatment with GPN was markedly reduced (Fig. 1F), suggesting pre-emptying or release of lysosomal Ca^2+^ after PA treatment, similar to the results using other types of cells [12, 17].

When we determined [Ca^2+^]_i_ and studied whether release of lysosomal Ca^2+^ leads to increased cytosolic Ca^2+^ content ([Ca^2+^]_i_), [Ca^2+^]_i_ estimated by Fluo-3 AM staining was significantly increased by PA (Fig. 1G), supporting release of lysosomal Ca^2+^ to the cytosol. When ratiometric measurement of [Ca^2+^]_i_ after Fura-2 staining was conducted to avoid interference due to uneven loading or photobleaching [23], increased [Ca^2+^]_i_ by PA treatment was again well observed, which was reversed by MitoTEMPO (Fig. 1G, H), indicating that mitochondrial ROS-induced lysosomal Ca^2+^ release leads to increased [Ca^2+^]_i_. Since molecules other than Ca^2+^ might be released from lysosome and could affect cell viability, we studied effect of an inhibitor of cathepsin B that has been reported to be released from hepatocytes after PA treatment and to induce lipotoxic cell death [24]. Ca-074Me, a cell-permeable inhibitor of cathepsin B, did not inhibit cell death by PA, suggesting no role of lysosomal cathepsin B release in lipotoxic cell death of HepG2 cells (Fig. 1I). Taken together, these data demonstrate that PA-induced mitochondrial ROS leads to release of lysosomal Ca^2+^ to cytoplasm and increased [Ca^2+^]_i_, contributing to lipotoxic HepG2 cell death.

### mPT pore (mPTP) opening mediates lysosomal Ca^2+^ loss-driven cell death in response to PA

**Fig. 2:**
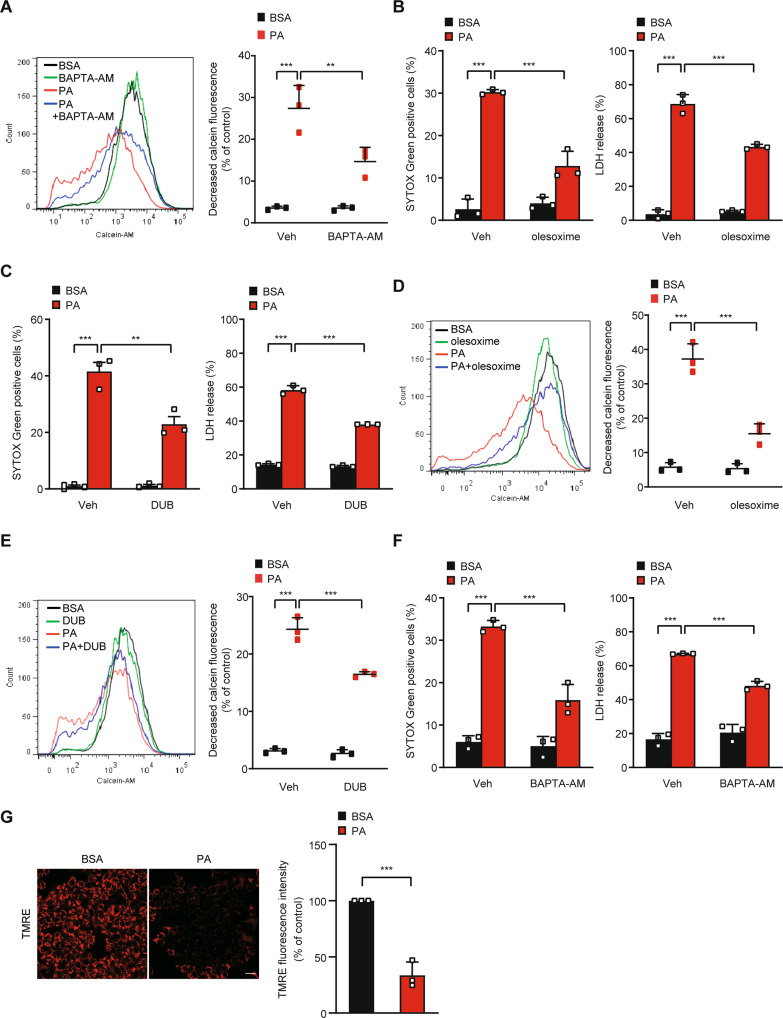
mPTP opening by PA due to lysosomal Ca^2+^ release. **A** After treatment of HepG2 cells with 500 μM PA in the presence or absence of 30 μM BAPTA-AM for 24 h, mPTP opening was assessed by flow cytometry after calcein-AM loading and Co^2+^ quenching (right). Representative histograms are shown (left). **B**, **C** After treatment of HepG2 cells with PA in the presence or absence of 100 μM olesoxime (**B**) or 200 μM DUB (**C**) for 24 h, cell death was determined using SYTOX Green (left) or LDH release assay (right). **D**, **E** After treatment of HepG2 cells with PA in the presence or absence of 100 μM olesoxime (**D**) or 200 μM DUB (**E**) for 24 h, mPTP opening was determined as in (**A**) (right). Representative histograms are shown (left). **F** After the same treatment as in (**A**), cell death was determined using SYTOX Green (left) or LDH release assay (right). **G** After treatment of HepG2 cells with PA as in (**A**), mitochondrial potential was determined by TMRE staining (right). Representative fluorescence images are shown (left). Scale bar, 20 μm. Data are expressed as the means ± SD of three independent experiments. All statistical comparisons were performed using one-way ANOVA with Tukey’s multiple comparison test. (*P < 0.05; **P < 0.01; ***P < 0.001; ns not significant).

We next studied whether PA-induced release of Ca^2+^ from lysosome into cytosol can induce mPTP opening because Ca^2+^ is one of the most important inducers of mPT which can lead to mitochondrial catastrophe and cell death [2, 25]. When cells were stained by calcein-AM/CoCl_2_ staining, mPTP opening visualized by Co^2+^ quenching of mitochondrial matrix calcein fluorescence [26] was well detected after PA treatment of HepG2 cells, showing occurrence of mPT by PA (Fig. 2A). PA-induced mPT was abrogated by BAPTA-AM chelating intracellular Ca^2+^ (Fig. 2A), indicating Ca^2+^-dependent mPT after PA treatment. Since mPT can lead to cell death such as necrosis or apoptosis depending on the severity of mPT and cellular context [15, 27, 28], we studied the role of mPT in HepG2 cell death by PA using inhibitors of mPT. Indeed, mPT inhibitors such as olesoxime [29] or decylubiquinone (DUB) [14], significantly reduced PA-induced cell death assessed by SYTOX Green staining or LDH release assay (Fig. 2B, C). Reduced lipotoxic cell death by olesoxime or DUB was accompanied by significantly reduced mPT as evidenced by decreased Co^2+^ quenching of calcein fluorescence (Fig. 2D, E), indicating that Ca^2+^-mediated mPTP opening is an important mechanism of PA-induced lipotoxicity. PA-induced HepG2 cell death was also significantly reduced by BAPTA-AM, confirming the role of increased [Ca^2+^]_i_ and Ca^2+^-mediated mPTP in lipotoxicity of HepG2 cells (Fig. 2F). As a consequence of mitochondrial damage associated with mPT, mitochondrial potential determined by TMRE staining was significantly lowered by PA treatment of HepG2 cells (Fig. 2G).

### ER → lysosome calcium flux and SOCE in lipotoxicity

**Fig. 3:**
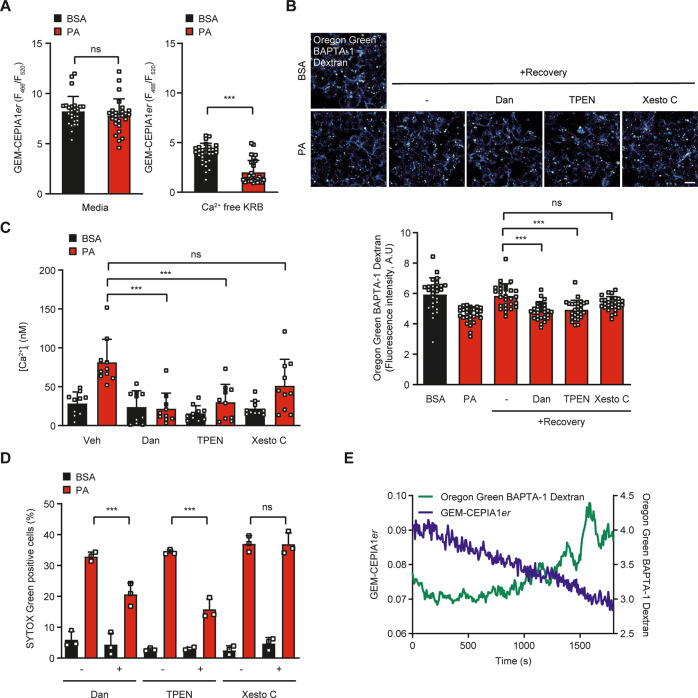
Role of ER → lysosome Ca^2+^ refilling in lipotoxicity. **A** After treatment of *GEM-CEPIA1er*-transfected HepG2 cells with PA for 6 h in a Ca^2+^-free KRBB (right) or a full medium (left), [Ca^2+^]_ER_ was determined by confocal microscopy. **B** After treatment of OGBD-labeled cells with PA for 6 h, cells were incubated in a fresh medium without PA in the presence or absence of 10 μM dantrolene (Dan), 10 μM TPEN or 3 μM Xestospongin C (Xesto C). Recovery of [Ca^2+^]_Lys_ after removal of PA was determined by confocal microscopy (lower). Representative fluorescence images are shown (upper). Scale bar, 20 μm. **C** After the same treatment as in B, [Ca^2+^]_i_ was determined by radiometric analysis after Fura-2 loading. **D** After the same treatment as in (**B**), cell death was evaluated by SYTOX Green staining. **E** After treatment of *GEM-CEPIA1er* transfected cells loaded with OGBD with PA for 6 h and washout, recovery of [Ca^2+^]_Lys_ and changes of [Ca^2+^]_ER_ were monitored simultaneously in the absence of extracellular Ca^2+^. Data are expressed as the means ± SD of three independent experiments. Statistical comparisons were performed using two-tailed Student’s *t*-test (**A**) or one-way ANOVA with Tukey’s multiple comparison test (**B**–**D**). (*P < 0.05; **P < 0.01; ***P < 0.001; ns not significant).

While we showed the role of lysosomal Ca^2+^ release in lipotoxicity, lysosomal Ca^2+^ might not be a sufficient source of Ca^2+^ required for full execution of cellular process requiring Ca^2+^, since lysosomal volume is 1% of cell volume and lysosomal Ca^2+^ pool is a relatively small Ca^2+^ reservoir [30]. We thus wondered whether release or emptying of lysosomal Ca^2+^ content in HepG2 cells treated with PA could be replenished from endoplasmic reticulum (ER), the largest intracellular Ca^2+^ reservoir [31] which has been observed when lysosomal Ca^2+^ emptying occurs (i.e., ER → lysosome Ca^2+^ refilling) [12, 31]. When [Ca^2+^]_ER_ was determined after PA treatment of HepG2 cells using *GEM-CEPIA1er* [32] in a Ca^2+^-free Krebs-Ringer bicarbonate (KRB) buffer to abolish possible store-operated Ca^2+^ entry (SOCE) [33], Ca^2+^ concentration in the ER ([Ca^2+^]_ER_) was significantly reduced (Fig. 3A), suggesting that ER Ca^2+^ is mobilized probably to replenish lysosomal Ca^2+^ loss. When [Ca^2+^]_ER_ was determined without removal of extracellular Ca^2+^, [Ca^2+^]_ER_ was not significantly reduced likely due to activation of store-operated Ca^2+^ entry (SOCE) after ER Ca^2+^emptying (Fig. 3A), which suggests that SERCA was not inhibited by PA since SERCA inhibition reduces [Ca^2+^]_ER_ regardless of extracellular Ca^2+^ [32] and eliminates the possibility of increased [Ca^2+^]_i_ after PA treatment due to SERCA inhibition.

Since these results suggested replenishment of lysosomal Ca^2+^ depletion by ER Ca^2+^, we next studied whether ER to lysosomal Ca^2+^ movement indeed occurs after PA treatment inducing lysosomal Ca^2+^ release. When PA was removed after treatment for 6 h, a decrease of [Ca^2+^]_Lys_ by PA treatment was recovered (Fig. 3B). To study the role of ER Ca^2+^ in the recovery of reduced [Ca^2+^]_Lys_, we studied the effect of blockade of ER Ca^2+^ exit channels that could be routes of ER to lysosomal Ca^2+^ movement during the recovery of [Ca^2+^]_Lys_. When we employed Xestospongin C, an IP3R antagonist, recovery of decreased [Ca^2+^]_Lys_ after removal of PA was not significantly affected (Fig. 3B). On the other hand, dantrolene, an antagonist of ryanodine receptor (RyR), another ER Ca^2+^ exit channel, significantly suppressed the recovery of suppressed [Ca^2+^]_Lys_ after removal of PA (Fig. 3B), suggesting involvement of RyR channel in ER → lysosome Ca^2+^ refilling after lysosome Ca^2+^ release by PA. When we chelated ER Ca^2+^ with a membrane-permeant metal chelator *N,N,N’,N’*-tetrakis (2-pyridylmethyl) ethylene diamine (TPEN) that has a low Ca^2+^ affinity and can chelate ER Ca^2+^ but not cytosolic Ca^2+^ [34], recovery of [Ca^2+^]_Lys_ after removal of PA was significantly inhibited (Fig. 3B), again supporting the role of ER Ca^2+^ in the recovery of lysosomal Ca^2+^.

We next studied the effect of blockade of ER Ca^2+^ exit channels on the increase of [Ca^2+^]_i_ after PA treatment. Again, dantrolene but not Xestospongin C, significantly suppressed the increase of [Ca^2+^]_i_ after PA treatment determined by ratiometric measurement following Fura-2 loading (Fig. 3C), suggesting that ER → lysosome Ca^2+^ refilling through RyR channel contributes to the increase of [Ca^2+^]_i_ after PA treatment by replenishing decreased lysosomal Ca^2+^ content and sustaining lysosomal Ca^2+^ release. TPEN that reduced recovery of decreased [Ca^2+^]_Lys_ also attenuated increase of [Ca^2+^]_i_ after PA treatment (Fig. 3C). When cell death was determined, dantrolene but not Xestospongin C, significantly suppressed the cell death after PA treatment (Fig. 3D), suggesting that ER → lysosome Ca^2+^ refilling through RyR channel contributes to the PA-induced cell death probably by supporting continuous increase of cytosolic Ca^2+^ and subsequent mPTP opening. TPEN also alleviated HepG2 cell death by PA (Fig. 3D), substantiating supportive role of ER Ca^2+^ in lipotoxicity. To study dynamic changes of [Ca^2+^]_ER_ and its temporal relationship with lysosomal Ca^2+^ refilling, we simultaneously traced [Ca^2+^]_ER_ and [Ca^2+^]_Lys_ in cells transfected with *GEM-CEPIA1er* and loaded with OGBD. When organelle [Ca^2+^] was monitored in cells that have reduced [Ca^2+^]_Lys_ after PA treatment for 6 h and subsequently were incubated in a Ca^2+^-free medium, a decrease of [Ca^2+^]_ER_ occurred in parallel with an increase of [Ca^2+^]_Lys_ (Fig. 3E), which strongly supports that ER → lysosome Ca^2+^ refilling occurs during HepG2 cell lipotoxicity.

**Fig. 4:**
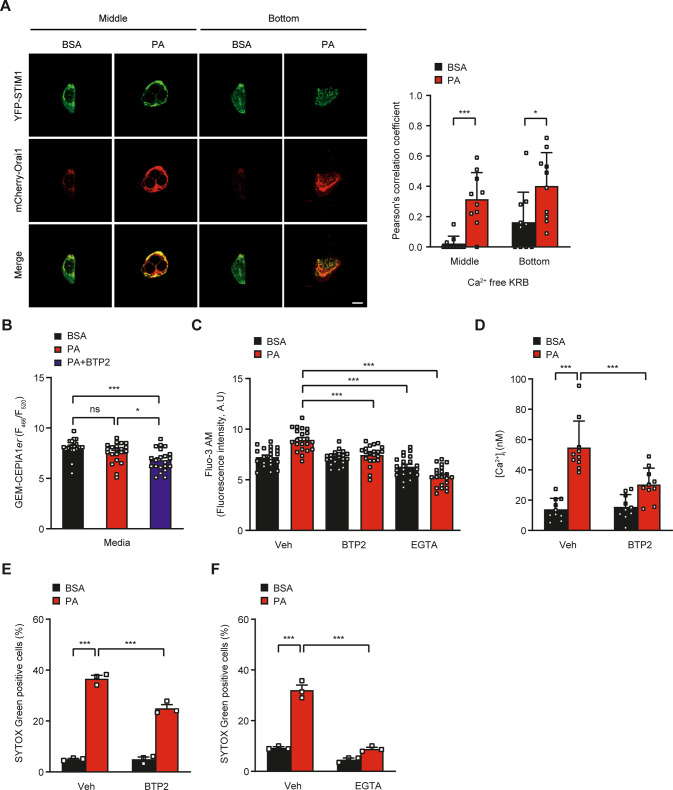
SOCE in lipotoxicity. **A** After treatment of HepG2 cells transfected with *YFP-STIM1* and *mCherry-Orai1* with PA for 6 h, STIM1 aggregation and its co-localization with ORAI1 were examined at the middle and bottom of the cells by confocal microscopy (left). Scale bar, 10 μm. Pearson’s correlation coefficient was calculated as an index of co-localization of YFP-STIM1 and mCherry-Orai1 (right). **B** After treatment of *GEM-CEPIA1er*-transfected cells with PA in the presence or absence of 10 μM BTP2 for 6 h, [Ca^2+^]_ER_ was determined by confocal microscopy. **C**, **D** After the same treatment as in (**B**), [Ca^2+^]_i_ was determined by confocal microscopy after Fluo-3 AM loading (**C**) or ratiometric analysis after Fura-2 loading (**D**). **E**, **F** After treatment of cells with PA in the presence or absence of 10 μM BTP2 (**E**) or 2 mM EGTA (**F**) for 24 h, cell death was evaluated using SYTOX Green. Data are expressed as the means ± SD of three independent experiments. Statistical comparisons were performed using two-tailed Student’s *t*-test (**A**) or one-way ANOVA with Tukey’s multiple comparison test (**B**–**F**). (*P < 0.05; **P < 0.01; ***P < 0.001; ns not significant).

Decrease of [Ca^2+^]_ER_ after PA treatment only in the absence of extracellular Ca^2+^ suggested SOCE without SERCA inhibition because SERCA inhibition would decrease [Ca^2+^]_ER_ regardless of extracellular Ca^2+^ [32]. Thus, we next studied possible occurrence and role of SOCE in lipotoxicity by PA. Since ER luminal protein STIM1 oligomerizes and recruits plasma membrane SOCE channel ORAI1 to activate Ca^2+^ entry when ER Ca^2+^ stores are reduced [33], we studied expression pattern of STIM1 after PA treatment that reduced ER Ca^2+^ store due to ER → lysosome Ca^2+^ refilling. In control-treated cells, neither STIM1 oligomerization nor co-localization between STIM1 and ORAI1 was observed (Fig. 4A). In contrast, both STIM1 oligomerization and co-localization between STIM1 and ORAI1 were well observed after PA treatment (Fig. 4A), supporting that reduced ER Ca^2+^ content after PA treatment induced SOCE activation. To validate the role of SOCE in intracellular Ca^2+^ flux, we studied the effect of BTP2, a blocker of SOCE [35, 36] on [Ca^2+^]_ER_. Indeed, in the presence of BTP2, [Ca^2+^]_ER_ was significantly reduced by PA even without removal of extracellular Ca^2+^. In contrast, without BTP2, PA-induced reduction of [Ca^2+^]_ER_ was not observed in the presence of extracellular Ca^2+^ (Fig. 4B), suggesting the occurrence of SOCE after PA treatment likely due to ER → lysosome Ca^2+^ refilling and decreased [Ca^2+^]_ER_ unrelated to SERCA inhibition. Furthermore, the increase of [Ca^2+^]_i_ by PA determined by Fluo-3 AM staining or ratiometric measurement after Fura-2 loading was significantly attenuated by BTP2 (Fig. 4C, D), indicating the role of SOCE in the increase of [Ca^2+^]_i_ by PA. Furthermore, PA-induced death of HepG2 cells was significantly reduced by BTP2 (Fig. 4E), supporting the role of SOCE in Ca^2+^-mediated lipotoxicity. EGTA chelating extracellular Ca^2+^ also significantly reduced increase of [Ca^2+^]_i_ and death of HepG2 cells after PA treatment (Fig. 4C, F), further supporting the role of extracellular Ca^2+^ flux in the increase of [Ca^2+^]_i_ and subsequent lipotoxicity.

### In vivo lipotoxicity

**Fig. 5:**
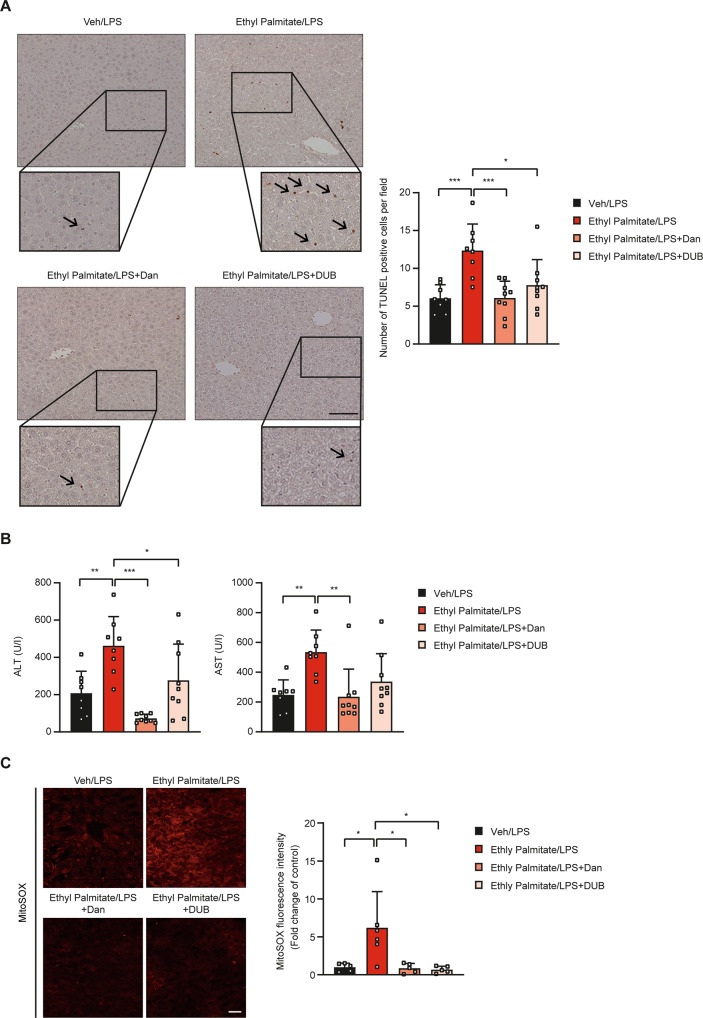
In vivo lipotoxicity. **A** After injection of ethyl palmitate and LPS to C57BL/6 mice with or without pretreatment with dantrolene or DUB, cell death was evaluated by TUNEL staining of hepatic sections (right). Representative TUNEL staining is presented (left) (*n* = 8–9/group). Insets were magnified. Scale bar, 200 μm. **B** In serum from mice of (**A**), ALT and AST levels were determined using a blood chemistry analyzer (*n* = 8–9/group). **C** In frozen hepatic sections from the mice of (**A**), mitochondrial ROS accumulation was determined by MitoSOX staining (*n* = 5–6/group) (right). Representative fluorescence images are shown (left). Scale bar, 20 μm. Data are presented as the means ± SD. All statistical comparisons were performed using one-way ANOVA with Tukey’s multiple comparison test. (*P < 0.05; **P < 0.01; ***P < 0.001; ns not significant).

Based on our in vitro results showing the role of lysosomal Ca^2+^ release coupled with ER → lysosome Ca^2+^ refilling in mPT and cell death in vitro, we next studied the effect of blockade of lysosomal Ca^2+^-mediated mPT on lipotoxicity in vivo. When mice were injected with ethyl palmitate followed by LPS administration, death of hepatocytes was observed by TUNEL staining which was accompanied by elevated serum alanine aminotransferase (ALT)/aspartate aminotransferase (AST) (Fig. 5A, B), similar to a previous paper [37]. When dantrolene that was able to inhibit PA-induced hepatocyte death through blockade of the increase of [Ca^2+^]_i_ was administered to mice before ethyl palmitate injection, death of hepatocytes identified by TUNEL staining was significantly ameliorated, suggesting blockade of in vivo lipotoxicity by dantrolene (Fig. 5A). Elevated serum ALT/AST levels after ethyl palmitate followed by LPS administration were also significantly reduced by dantrolene pretreatment (Fig. 5B). In addition, accumulation of mitochondrial ROS stained by MitoSOX was observed in the liver tissue of mice treated with LPS + ethyl palmitate (Fig. 5C), which is in line with mitochondrial ROS accumulation in HepG2 cells treated with PA in vitro. Such mitochondrial ROS accumulation in vivo was ameliorated by dantrolene administration (Fig. 5C). We also studied whether blockade of mPT could inhibit lipotoxicity in vivo based on our in vitro results showing the role of mPT in PA-induced lipotoxicity. When we pretreated mice with DUB, an inhibitor of mPT, hepatocyte death detected by TUNEL staining or elevated serum ALT/AST levels was significantly reduced (Fig. 5A, B), indicating the role of mPT in lipotoxicity in vivo. Mitochondrial ROS in the liver tissue of mice treated with LPS + ethyl palmitate was also reduced by DUB (Fig. 5C). These results showing amelioration of PA-induced hepatocyte death in vivo by dantrolene or DUB, suggest that lipotoxicity of hepatocytes in vivo can be mediated by Ca^2+^-mediated mPT and indicate the role of lysosomal Ca^2+^ release supported by ER → lysosome Ca^2+^ refilling and SOCE in mPT-induced lipotoxicity observed in vitro.

## Discussion

It is well established that PA, as an effector of metabolic stress in vitro, can induce stress in diverse organelles such as ER and mitochondria [2, 4, 8, 9]. However, recent investigation showed that PA can induce stress or dysfunction of lysosome as well [10–13], such as elevated pH or reduced Ca^2+^ content due to release of Ca^2+^ from lysosome. Increased cytosolic Ca^2+^ can mediate diverse beneficial or harmful effects on cells. Since Ca^2+^ is a well-known inducer of mPT [14, 15], we hypothesized that lysosomal Ca^2+^ release might contribute to cell death after treatment with PA or lipotoxicity. Indeed, we observed that Ca^2+^ could be released from lysosome after PA treatment, which is likely due to activation of lysosomal Ca^2+^ exit channel by mitochondrial ROS. After release of lysosomal Ca^2+^, [Ca^2+^]_i_ was increased, which imposed mPT and subsequent death. Cell death due to mPT caused by PA-induced lysosomal Ca^2+^ release was inhibited by mPT inhibitors such as olesoxime or DUB, showing the role of lysosomal Ca^2+^ release and consequent mPT in lipotoxicity, which is consistent with previous papers reporting mPT induction by PA [2, 38]. Identity of lysosomal Ca^2+^ exit channel responsible for PA-induced lysosomal Ca^2+^ release is not clear. Previous papers have reported the role of TRPML1 or TRPM2 channel in Ca^2+^- or Zn^2+^-mediated cell death [39–41]. We have also studied the effect of inhibitors of TRPML1 or TRPM2. However, ML-SI1, ML-SI3 or N-(p-amylcinnamoyl)anthranilic acid inhibiting TRPML1, TRPML1/2/3 and TRPM2, respectively [42–44], did not inhibit PA-induced lipotoxic death of HepG2 cells (Oh S-J et al., unpublished results). Furthermore, Ned-19, an inhibitor of TPC1/2 which has been reported to be associated with ischemia-reperfusion injury [45], was also without effect (Oh S-J et al., unpublished results). Besides such lysosomal Ca^2+^ exit channels, other members of the TRPM family or those belonging to different families might play a role in Ca^2+^-mediated lipotoxic cell death, which could be a subject for further studies. It remains to be clarified which lysosomal Ca^2+^ channel is involved in lipotoxicity due to lysosomal Ca^2+^-mediated mPT.

It has been demonstrated that ER → lysosome Ca^2+^ flux occurs when lysosomal Ca^2+^ content is lowered due to lysosomal Ca^2+^ release by mitochondrial stressors [12, 31]. Such refilling of lysosomal Ca^2+^ pool from ER Ca^2+^ pool occurring when lysosomal Ca^2+^ content is lowered, is likely due to small lysosomal volume accommodating Ca^2+^ which is 10% of ER Ca^2+^ volume [30]. Thus, while [Ca^2+^]_Lys_ is comparable to [Ca^2+^]_ER_, lysosomal Ca^2+^ content might not be sufficient for progression of events requiring Ca^2+^ flux. Besides ER Ca^2+^ pool, another source of lysosomal Ca^2+^ could be endocytic pathway. However, most of endocytic Ca^2+^ has been reported to be dissipated before reaching lysosome [46], supporting the importance of ER Ca^2+^ as a dominant source of lysosomal Ca^2+^ pool. The role of ER → lysosome Ca^2+^ flux in diverse pathological and physiological conditions could be an intriguing topic to be explored in future studies. When we studied whether similar phenomenon occurs after PA treatment of HepG2 cells, ER → lysosome Ca^2+^ refilling could be clearly demonstrated by simultaneous monitoring of [Ca^2+^]_ER_ and [Ca^2+^]_Lys_ during incubation in a Ca^2+^-free medium ensuring recovery of [Ca^2+^]_Lys_ after removal of PA. Furthermore, blockade of ER → lysosome Ca^2+^ refilling with dantrolene, an inhibitor of RyR Ca^2+^ exit channel on ER or chelation of ER Ca^2+^ by TPEN could inhibit lipotoxicity, which indicates that intracellular Ca^2+^ flux from ER occurs to sustain lysosomal Ca^2+^ release, resulting in cell death. Ca^2+^ flux from ER to lysosome, in turn, induced ER Ca^2+^ emptying which activated SOCE. Occurrence of SOCE after PA treatment of HepG2 cells was evidenced by no decrease of [Ca^2+^]_ER_ in cells treated with PA without removal of extracellular Ca^2+^, reappearance of the decrease of [Ca^2+^]_ER_ by BTP2 even in the presence of extracellular Ca^2+^ and STIM1 aggregation co-localized with ORAI1. Functional role of SOCE after treatment with PA was demonstrated by inhibition of lipotoxicity by BTP2 or extracellular Ca^2+^ chelation with EGTA. Furthermore, occurrence of SOCE and no decrease of [Ca^2+^]_ER_ in cells treated with PA without removal of extracellular Ca^2+^ supports that increase of [Ca^2+^]_i_ after PA treatment is not due to direct release of Ca^2+^ from ER through SERCA inhibition, since increase [Ca^2+^]_i_ due to SERCA inhibition would not be affected by extracellular Ca^2+^ [32]. While SERCA inhibition is an important component of ER stress associated with lipid overload or obesity in vivo inducing altered membrane phospholipid composition [47], SERCA might not be important in acute lipotoxicity of HepG2 cells in vitro. However, we do not eliminate the possibility that ER stress might contribute to lipotoxicity in vitro. In fact, we have observed that CHOP, an important player in ER stress-induced cell death, can be activated by mitochondrial ROS produced after PA treatment [48].

In our experiments to investigate the role of increased Ca^2+^ and mPT in lipotoxicity in vivo, we observed that DUB inhibiting mPT and dantrolene abrogating [Ca^2+^]_i_ increase and cell death in vitro by PA could inhibit lipotoxic death of hepatocytes in vivo. Reduction of mitochondrial ROS by dantrolene or DUB on mitochondrial ROS could be due to a feed-forward response leading to further ROS release following Ca^2+^-mediated mPT [49, 50] which was inhibited by dantrolene or DUB. Dantrolene is a candidate for therapeutic drug against malignant hyperthermia or Alzheimer’s disease [51, 52]. DUB has also been studied as a potential therapeutic agent against Friedreich’s ataxia [53] or tumor-induced angiogenesis [54]. Our results suggest the possibility that dantrolene or DUB could be considered candidates for drug agents against diseases characterized by lipotoxicity such as nonalcoholic steatohepatitis or cardiomyopathy. While we have shown the contribution of mPT induced by lysosomal Ca^2+^ release as a mechanism of lipotoxicity in vitro and in vivo, other previously reported mechanisms of lipotoxicity such as ER or mitochondrial stress and generation of ceramide or lysophosphatidylcholine [1–4] could also play an important role depending on the cellular context or environmental condition, and optimal strategy against lipotoxicity could be different accordingly.

## Materials and methods

### Reagents

Reagents in this study were purchased from following sources: Oregon Green-488 BAPTA-1 dextran (OGBD, O6789), Fluo-3 AM (F23915), MitoSOX^TM^ Red (M36008), SYTOX Green (S7020), BAPTA-AM (O6807), calcein-AM (C1430), Fura-2 (F1221) from Thermo Fisher Scientific; palmitate (PA, P0500), decylubiquinone (DUB, D7911), bovine serum albumin (BSA, A9418), cobalt chloride (CoCl_2_, 232696), ethylene glycol-bis (2-aminoethylether)-N,N,N′,N′-tetraacetic acid (EGTA, 03777), MitoTEMPO (SML0737), CA-074Me (205531) from Sigma-Aldrich; Gly-Phe β-naphthylamide (GPN, ab145914) from Abcam; BTP2 (S8380) from Selleckchem; CytoTox 96® Non-Radioactive Cytotoxicity Assay (G1780) from Promega^TM^ Corporation.

### Cell culture and treatment

HepG2 cells obtained from Korean Cell Line Bank (KCLB) were grown in DMEM medium (Welgene, LM-001-05)-1% penicillin–streptomycin–amphotericin B mixture (Lonza, 17-745E) supplemented with 10% fetal bovine serum (Corning, 35-010-CV). Cells were tested for mycoplasma contamination using a Mycoplasma PCR Detection Kit (e-MycoTM, 25236, iNtRON Biotechnology). PA stock solution was prepared by dissolving palmitate in 70% ethanol and heating at 56 °C. PA stock solution was diluted in 2% fatty acid-free BSA-DMEM before treatment. For in vitro treatment, following concentrations were employed: OGBD, 100 μg/ml; Fluo-3 AM, 5 μM; MitoSOX^TM^ Red, 5 μM; SYTOX Green Nucleic Acid Stain, 1 μM; BAPTA-AM, 30 μM; calcein-AM, 1 μM; palmitic acid (PA), 500 μM; decylubiquinone (DUB), 200 μM; cobalt chloride (CoCl_2_), 2 mM; Gly-Phe β-naphthylamide (GPN), 200 μM; BTP2, 10 μM; MitoTEMPO, 10 μM; EGTA, 2 mM; CA-074Me, 10 μM.

### SYTOX Green nucleic acid staining

Cell death was evaluated using SYTOX^TM^ Green Nucleic Acid Stain kit (Thermo Fisher Scientific, S7020). Briefly, HepG2 cells were seeded in a 24-well plate. After 24 h, cells were treated with PA with or without indicated compound for 24 h. Cells were then incubated with SYTOX Green (1 μM) for 30 min at 37 °C. SYTOX Green fluorescence was measured by flow cytometry using BD FACSVerse and FACSCanto II (BD Biosciences, San Jose, CA, USA). Data analysis was performed using FlowJo software (10.8, FlowJo, LLC, BD Biosciences).

### LDH release assay

Cell death was assessed using an LDH release assay kit (Promega^TM^ Corporation, G1780). Briefly, HepG2 cells seeded 4 × 10^4^ well in a 96-well plate were treated with PA with or without indicated compound for 24 h. Culture supernatant was collected and analyzed according to the manufacturer’s protocol.

### Transfection and plasmids

Cells were transfected with plasmids such as *GCaMP3-ML1*, *GEM-CEPIA1er*, *YFP-STIM1*, *3xFLAG-mCherry Red-Orai1/P3XFLAG7.1* using PolyJet^TM^ In Vitro DNA Transfection Reagent (SigmaGen^®^ Laboratories, SL100688), according to the manufacturer’s protocol.

### Measurement of cytosolic, ER and lysosome Ca^2+^ contents

To measure [Ca^2+^]_Lys_, HepG2 cells grown on a chambered coverglass (Thermo Fisher Scientific, 155383) were loaded with 100 μg/ml OGBD, an indicator of lysosomal luminal Ca^2+^ for 16 h. After incubation in a fresh media for 4 h, cells were treated with BSA or 500 μM PA for 6 h. After washing with Ca^2+^-free HEPES-buffered saline (HBS), fluorescence was measured using an LSM780 confocal microscope (Zeiss, Oberkochen, Germany) and quantified using ImageJ software.

To determine [Ca^2+^]_i_ by confocal microscopy, HepG2 cells grown on chambered coverglass were loaded with 5 μM Fluo-3 AM for 30 min, an indicator of cytosolic Ca^2+^. After treatment with 500 μM PA for 6 h, cells were washed with Ca^2+^-free PBS twice, and fluorescence was recorded using an LSM780 confocal microscope. Fluorescence intensity was quantified using ImageJ software. For ratiometric [Ca^2+^]_i_ measurement, cells were loaded with 2 μM of the acetoxymethyl ester form of Fura-2 in DMEM at 37 °C for 30 min and then washed with a basal Ca^2+^ solution (145 mM NaCl, 5 mM KCl, 3 mM MgCl_2_, 10 mM glucose, 1 mM EGTA, 20 mM HEPES, pH 7.4). Measurements were conducted using MetaFluor on an Axio Observer A1 (Zeiss) equipped with a 150 W xenon lamp Polychrome V (Till Photonics, Bloaaom Drive Victor, NY, USA), a CoolSNAP-Hq2 digital camera (Photometrics, Tucson, AZ, USA), and a Fura-2 filter set. Fluorescence at 340/380 nm was measured in phenol red-free medium, and converted to [Ca^2+^]_i_ using the following equation [23].

$$\left[ {{{{\mathrm{Ca}}}}^{2 + }} \right]_{{{\mathrm{i}}}} = {{{\mathrm{K}}}}_d \times \left[ {\left( {R - R_{\min }} \right)/\left( {R_{\max } - R} \right) \times } \right.\left[ {F_{\min (380)}/F_{\max (380)}} \right]$$

where K_d_ = Fura-2 dissociation constant (224 nM at 37 °C), *F*_min(380)_ = the 380 nm fluorescence in the absence of Ca^2+^, *F*_max(380)_ = 380 nm fluorescence with saturating Ca^2+^, *R* = 340/380 nm fluorescence ratio, *R*_max_ = 340/380 nm ratio with saturating Ca^2+^, and *R*_min_ = 340/380 nm ratio in the absence of Ca^2+^.

To determine ER Ca^2+^ contents ([Ca^2+^]_ER_), HepG2 cells were grown on chambered coverglass and transfected with a *GEM-CEPIA1er* plasmid, a ratiometric fluorescent indicator of ER Ca^2+^. After 24 h, cells were treated with BSA or 500 μM PA for 6 h, and then washed with Ca^2+^-free KRBB (Sigma-Aldrich, K4002). GEM-CEPIA1*er* fluorescence was measured using an LSM780 confocal microscope at an excitation wavelength of 405 nm and an emission wavelength of 466 or 520 nm. Fluorescence ratio F466/F520 was calculated as an index of [Ca^2+^]_ER_ [32].

### GCaMP3-ML1 Ca^2+^ imaging

To detect perilysosomal Ca^2+^ release, HepG2 cells were transfected with a *GCaMP3-ML1* Ca^2+^ probe, a lysosome-targeted genetically-encoded Ca^2+^ sensor. Twenty-four h after transfection, cells were treated with BSA or 500 μM PA for 6 h. Perilysosomal Ca^2+^ release was recorded by monitoring GCaMP3 fluorescence intensity at 470 nm in a basal Ca^2+^ solution (145 mM NaCl, 5 mM KCl, 3 mM MgCl_2_, 10 mM glucose, 1 mM EGTA, 20 mM HEPES, pH 7.4) using a Zeiss LSM780 confocal microscope. GPN, a lysosomotropic agent, of 200 μM was added to evoke lysosomal Ca^2+^ release. GCaMP3-ML1 fluorescence was calculated as a change GCaMP3 fluorescence (ΔF) over baseline fluorescence (F_0_).

### STIM1 and ORAI1 co-localization

After transfection with *YFP-STIM1* and *3xFLAG-mCherry Red-Orai1/P3XFLAG7.1* (provided by Yuan J through Cha S-G) plasmids for 48 h, HepG2 cells were treated with BSA or 500 μM PA for 6 h. Cells were then fixed with 4% paraformaldehyde (PFA) at room temperature for 10 min. Fluorescence images were acquired with an LSM780 confocal microscope, and the co-localization between STIM1 and ORAI1 was examined by calculating Pearson’s correlation coefficient using ZEN software (Carl Zeiss Microscopy GmbH, Jena, Germany).

### Measurement of mitochondrial ROS

Mitochondrial ROS was measured using MitoSOX^TM^ Red. Briefly, HepG2 cells were pretreated with 10 μM MitoTEMPO for 1 h, and then treated with 500 μM PA for 24 h. After incubation with 5 μM MitoSOX^TM^ Red at 37 °C for 15 min, fluorescence was measured by flow cytometry using BD FACSVerse or FACSCanto II. Data were analyzed using FlowJo software.

To determine mitochondrial ROS accumulation in vivo, frozen sections of the liver tissue were incubated with MitoSOX^TM^ Red at 37 °C for 15 min, and then washed with PBS. Fluorescence images were observed with an LSM710 confocal microscope and fluorescence intensity was quantified using ImageJ software (NIH, Bethesda, MD, USA).

### mPTP opening

mPTP opening was assessed by Co^2+^ quenching of calcein-AM fluorescence. Cells were treated with PA (500 μM) with or without DUB (200 μM) or olesoxime (100 μM) for 24 h. Cells were then loaded with calcein-AM (1 μM) and CoCl_2_ (2 mM) at 37 °C for 15 min. Calcein fluorescence was measured by flow cytometry employing BD FACSVerse and FACSCanto II and analyzed using FlowJo software.

### Mitochondrial membrane potential

Mitochondrial membrane potential was determined using tetramethylrhodamine ethyl ester perchlorate (TMRE). After treatment of HepG2 cells with PA for 24 h, cells were incubated with 100 nM TMRE at 37 °C for 30 min, and fluorescence was measured using LSM710 confocal microscope. Fluorescence intensity was quantified using ImageJ software.

### Animals

Eight-week-old C57BL/6N male mice were purchased from Orient Bio (Seongnam, Korea). All experiments using mice were performed in accordance with the guidelines of the Public Health Service Policy in Humane Care and Use of Laboratory Animals. The protocol was approved by the Institutional Animal Care and Use Committee (IACUC) of the Department of Laboratory Animal Resources of Soonchunhyang Institute of Medi-bio Science. Ethyl palmitate (Tokyo Chemical Industry, P0003) was dissolved in water with 4.8% lecithin (FUJIFILM Wako Pure Chemical Corporation, 120-00832) and 10% glycerol (Sigma-Aldrich, G2025) to make a mixture containing ethyl palmitate at a concentration of 300 mM. LPS (Sigma-Aldrich, E. coli O55:B5) was dissolved in PBS, and 0.025 mg/kg LPS was injected intraperitoneally into mice. Dantrolene and DUB were dissolved in DMSO and diluted with PBS. For in vivo administration, dantrolene (10 mg/kg), DUB (5 mg/kg) or DMSO solution diluted in PBS was injected into mice that were fasted for 24 h. After 1 h, 300 mM ethyl palmitate or vehicle was administered, followed by LPS injection 1 h later. Blood and liver tissue samples were obtained 24 h later.

### Blood chemistry

Serum ALT and AST levels were measured using a Fuji Dri-Chem NX500i chemistry Analyzer (Fujifilm, Tokyo, Japan).

### TUNEL staining

Paraffin-embedded liver tissue blocks were prepared by fixing in 10% neutral buffered formalin (Sigma, HT501128). TUNEL staining was conducted using In Situ Cell Death Detection kit (Roche, 11684817910) according to the manufacturer’s instructions. Cell death index was expressed as the number of TUNEL-positive cells per field counted from more than 20 fields randomly selected (×200).

### Statistical analysis

Data are presented as means ± SD of three independent experiments. Two-tailed Student’s *t*-test was used to compare values between two groups. One-way ANOVA with Tukey’s test was used to compare values between multiple groups. Data were considered significant when *P* < 0.05. Data analysis was performed using GraphPad Prism 8 software (GraphPad Software, La Jolla, CA, USA).

## Data Availability

The data generated or analyzed during the current study and materials are available from the corresponding author without imposed restriction on reasonable request.

## References

[1] Han MS, Park SY, Shinzawa K, Kim S, Chung KW, Lee JH (2008). Lysophosphatidylcholine as a death effector in lipoapoptosis of hepatocytes. J Lipid Res.

[2] Koshkin V, Dai FF, Robson-Doucette CA, Chan CB, Wheeler MB (2008). Limited mitochondrial permeabilization is an early manifestation of palmitate-induced lipotoxicity in pancreatic beta-cells. J Biol Chem.

[3] Shimabukuro M, Wang MY, Zhou YT, Newgard CB, Unger RH (1998). Protection against lipoapoptosis of b cells through leptin-dependent maintenance of Bcl-2 expression. Proc Natl Acad Sci USA.

[4] Sommerweiss D, Gorski T, Richter S, Garten A, Kiess W (2013). Oleate rescues INS-1E β-cells from palmitate-induced apoptosis by preventing activation of the unfolded protein response. Biochem Biophys Res Com.

[5] Katsoulieris E, Mabley JG, Samai M, Green IC, Chatterjee PK (2009). alpha-Linolenic acid protects renal cells against palmitic acid lipotoxicity via inhibition of endoplasmic reticulum stress. Eur J Pharmacol.

[6] Khan MJ, Alam MR, Waldeck-Weiermair M, Karsten F, Groschner L, Riederer M (2012). Inhibition of autophagy rescues palmitic acid-induced necroptosis of endothelial cells. J Biol Chem.

[7] Shimabukuro M, Zhou YT, Levi M, Unger RH (1998). Fatty acid-induced b cell apoptosis: a link between obesity and diabetes. Proc Natl Acad Sci USA.

[8] Sparagna GC, Hickson-Bick DL, Buja LM, McMillin JB (2000). A metabolic role for mitochondria in palmitate-induced cardiac myocyte apoptosis. Am J Physiol.

[9] Xu S, Nam SM, Kim JH, Das R, Choi SK, Nguyen TT (2015). Palmitate induces ER calcium depletion and apoptosis in mouse podocytes subsequent to mitochondrial oxidative stress. Cell Death Dis.

[10] Karasawa T, Kawashima A, Usui-Kawanishi F, Watanabe S, Kimura H, Kamata R (2018). Saturated fatty acids undergo intracellular crystallization and activate the NLRP3 inflammasome in macrophages. Arterioscler Thromb Vasc Biol.

[11] Kim J, Kim SH, Kang H, Lee S, Park S-Y, Cho Y (2021). TFEB-GDF15 axis protects against obesity and insulin resistance as a lysosomal stress response. Nature Metabolism.

[12] Park K, Lim H, Kim J, Hwang H, Lee YS, Bae SH (2022). Essential role of lysosomal Ca2+-mediated TFEB activation in mitophagy and functional adaptation of pancreatic β-cells to metabolic stress. Nat Commun.

[13] Trudeau KM, Colby AH, Zeng J, Las G, Feng JH, Grinstaff MW (2016). Lysosome acidification by photoactivated nanoparticles restores autophagy under lipotoxicity. J Cell Biol.

[14] Fontaine E, Ichas F, Bernardi P (1998). A ubiquinone-binding site regulates the mitochondrial permeability transition pore. J Biol Chem.

[15] Nakagawa T, Shimizu S, Watanabe T, Yamaguchi O, Otsu K, Yamagata H (2005). Cyclophilin D-dependent mitochondrial permeability transition regulates some necrotic but not apoptotic cell death. Nature.

[16] Schönfeld P, Wojtczak L (2007). Fatty acids as modulators of the cellular production of reactive oxygen species. Free Radic Biol Med.

[17] Zhang X, Cheng X, Yu L, Yang J, Calvo R, Patnaik S (2016). MCOLN1 is a ROS sensor in lysosomes that regulates autophagy. Nat Commun.

[18] Chang I, Cho N, Kim S, Kim JY, Kim E, Woo JE (2004). Role of calcium in pancreatic islet cell death by IFN-gamma/TNF-alpha. J Immunol.

[19] Maher P, van Leyen K, Dey PN, Honrath B, Dolga A, Methner A (2018). The role of Ca 2+ in cell death caused by oxidative glutamate toxicity and ferroptosis. Cell Calcium.

[20] Zhivotovsky B, Orrenius S (2011). Calcium and cell death mechanisms: a perspective from the cell death community. Cell Calcium.

[21] Hunnicutt JH, Hardy RW, Williford J, McDonald JM (1994). Saturated fatty acid-induced insulin resistance in rat adipocytes. Diabetes.

[22] Shen D, Wang X, Li X, Zhang X, Yao Z, Dibble S (2012). Lipid storage disorders block lysosomal trafficking by inhibiting a TRP channel and lysosomal calcium release. Nat Commun.

[23] Grynkiewicz G, Poenie M, Tsien RY (1985). A new generation of Ca2+ indicators with greatly improved fluorescence properties. J Biol Chem.

[24] Li Z, Berk M, McIntyre TM, Gores GJ, Feldstein AE (2008). The lysosomal-mitochondrial axis in free fatty acid-induced hepatic lipotoxicity. Hepatology.

[25] Rial E, Rodríguez-Sánchez L, Gallardo-Vara E, Zaragoza P, Moyano E, González-Barroso MM (2008). Lipotoxicity, fatty acid uncoupling and mitochondrial carrier function. Biochim Biophys Acta.

[26] Petronilli V, Miotto G, Canton M, Colonna R, Bernardi P, Di Lisa F (1998). Imaging the mitochondrial permeability transition pore in intact cells. Biofactors.

[27] Bradham CA, Qian T, Streetz K, Trautwein C, Brenner DA, Lemasters JJ (1998). The mitochondrial permeability transition is required for tumor necrosis factor alpha-mediated apoptosis and cytochrome c release. Mol Cell Biol.

[28] Halestrap AP, Kerr PM, Javadov S, Woodfield KY (1998). Elucidating the molecular mechanism of the permeability transition pore and its role in reperfusion injury of the heart. Biochim Biophys Acta.

[29] Bordet T, Berna P, Abitbol J-L, Pruss RM (2010). Olesoxime (TRO19622): a novel mitochondrial-targeted neuroprotective compound. Pharmaceuticals.

[30] Penny CJ, Kilpatrick BS, Han JM, Sneyd J, Patel S (2014). A computational model of lysosome-ER Ca2+ microdomains. J Cell Sci.

[31] Garrity AG, Wang W, COllier CMD, Levery SA, Gao Q, Xu H (2016). The endoplasmic reticulum, not the pH gradient, drives calcium refilling of lysosomes. eLife.

[32] Suzuki J, Kanemaru K, Ishii K, Ohkura M, Lino M (2014). Imaging intraorganellar Ca2+ at subcellular resolution using CEPIA. Nat Commun.

[33] Derler I, Jardin I, Romanin C (2016). Molecular mechanisms of STIM/Orai communication. Am J Physiol.

[34] Hofer AM, Fasolato C, Pozzan T (1998). Capacitative Ca2+ entry is closely linked to the filling state of internal Ca2+ stores: a study using simultaneous measurements of ICRAC and Intraluminal [Ca2+]. J Cell Biol.

[35] Bogeski I, Al-Ansary D, Qu B, Niemeyer BA, Hoth M, Peinelt C (2010). Pharmacology of ORAI channels as a tool to understand their physiological functions. Expert Rev Clin Pharmacol.

[36] Zitt C, Strauss B, Schwarz EC, Spaeth N, Rast G, Hatzelmann A (2004). Potent inhibition of Ca2+ release-activated Ca2+ channels and T-lymphocyte activation by the pyrazole derivative BTP2. J Biol Chem.

[37] Ogawa Y, Imajo K, Honda Y, Kessoku T, Tomeno W, Kato S (2018). Palmitate-induced lipotoxicity is crucial for the pathogenesis of nonalcoholic fatty liver disease in cooperation with gut-derived endotoxin. Sci Rep.

[38] Srivastava S, Chan C (2007). Hydrogen peroxide and hydroxyl radicals mediate palmitate-induced cytotoxicity to hepatoma cells: relation to mitochondrial permeability transition. Free Radic Res.

[39] Akyuva Y, Nazıroğlu M (2020). Resveratrol attenuates hypoxia-induced neuronal cell death, inflammation and mitochondrial oxidative stress by modulation of TRPM2 channel. Sci Rep.

[40] Du W, Gu M, Hu M, Pinchi P, Chen W, Ryan M (2021). Lysosomal Zn 2+ release triggers rapid, mitochondria-mediated, non-apoptotic cell death in metastatic melanoma. Cell Rep.

[41] Zaibi N, Li P, Xu S-Z (2021). Protective effects of dapagliflozin against oxidative stress-induced cell injury in human proximal tubular cells. PLoS ONE.

[42] Kraft R, Grimm C, Frenzel H, Harteneck C (2006). Inhibition of TRPM2 cation channels by N-(p-amylcinnamoyl)anthranilic acid. Br J Pharma.

[43] Samie M, Wang X, Zhang X, Goschka A, Li X, Cheng X (2013). A TRP channel in the lysosome regulates large particle phagocytosis via focal exocytosis. Dev Cell.

[44] Sun J, Liu Y, Hao X, Lin W, Su W, Chiang E (2022). LAMTOR1 inhibition of TRPML1-dependent lysosomal calcium release regulates dendritic lysosome trafficking and hippocampal neuronal function. EMBO J.

[45] Simon JS, Vrellaku B, Monterisi S, Chu SM, Rawlings N, Lomas O (2020). Oxidation of protein kinase A regulatory subunit PKARIα porotects against myocardial ischemia-reperfusion injury byinhibiting lysosomal-triggered calcium release. Circulation.

[46] Gerasimenko JV, Tepikin AV, Petersen OH, Gerasimenko OV (1998). Calcium uptake via endocytosis with rapid release from acidifying endosomes. Curr Biol.

[47] Fu S, Yang L, Li P, Hofmann O, Dicker L, Hide W (2011). Aberrant lipid metabolism disrupts calcium homeostasis causing liver endoplasmic reticulum stress in obesity. Nature.

[48] Ly LD, Ly DD, Nguyen NT, Kim J-H, Yoo H, Chung J (2020). Mitochondrial Ca2+ uptake relieves palmitate-induced cytosolic Ca2+ overload in MIN6 cells. Mol Cells.

[49] Zorov DB, Filburn CR, Klotz LO, Zweier JL, Sollott SJ (2000). Reactive oxygen species (ROS)-induced ROS release: a new phenomenon accompanying induction of the mitochondrial permeability transition in cardiac myocytes. J Exp Med.

[50] Zorov DB, Juhaszova M, Sollott SJ (2014). Mitochondrial reactive oxygen species (ROS) and ROS-induced ROS release. Physiol Rev.

[51] Karagas NE, Venkatachalam K (2019). Roles for the endoplasmic reticulum in regulation of neuronal calcium homeostasis. Cells.

[52] Kim KSM, Kriss RS, Tautz TJ (2019). Malignant hyperthermia: a clinical review. Adv Anesth.

[53] Jauslin ML, Meier T, Smith RA, Murphy MP (2003). Mitochondria-targeted antioxidants protect Friedreich Ataxia fibroblasts from endogenous oxidative stress more effectively than untargeted antioxidants. FASEB J.

[54] Cao J, Liu X, Yang Y, Wei B, Li Q, Mao G (2020). Decylubiquinone suppresses breast cancer growth and metastasis by inhibiting angiogenesis via the ROS/p53/ BAI1 signaling pathway. Angiogenesis.

